# Embelin inhibits TNF-α converting enzyme and cancer cell metastasis: molecular dynamics and experimental evidence

**DOI:** 10.1186/1471-2407-14-775

**Published:** 2014-10-22

**Authors:** Jaspreet Kaur Dhanjal, Nupur Nigam, Sudhanshu Sharma, Anupama Chaudhary, Sunil C Kaul, Abhinav Grover, Renu Wadhwa

**Affiliations:** School of Biotechnology, Jawaharlal Nehru University, New Delhi, 110 067 India; Cell Proliferation Research Group and DBT-AIST International Laboratory for Advanced Biomedicine, National Institute of Advanced Industrial Science & Technology (AIST), Central 4, 1-1-1 Higashi, Tsukuba, Ibaraki, 305 8562 Japan; Graduate School of Life and Environmental Sciences, University of Tsukuba, Tsukuba, Japan

**Keywords:** Embelin, Breast cancer cells, TACE inhibition, MMP inactivation, Anticancer

## Abstract

**Background:**

Embelin, a quinone derivative, is found in the fruits of *Embelia ribes* Burm (Myrsinaceae). It has been shown to have a variety of therapeutic potentials including anthelmintic, anti-tumor, anti-diabetic, anti-bacterial and anti-inflammation. Inflammation is an immunological response to external harmful stimuli and is regulated by an endogenous pyrogen and pleiotropic pro-inflammatory cytokine, tumor necrosis factor alpha (TNF-α). TNF-α production has been implicated in a variety of other human pathologies including neurodegeneration and cancer. Several studies have shown that the anti-inflammatory activity of embelin is mediated by reduction in TNF-α. The latter is synthesized as a membrane anchored protein (pro-TNF-α); the soluble component of pro-TNF-α is then released into the extracellular space by the action of a protease called TNF-α converting enzyme (TACE). TACE, hence, has been proposed as a therapeutic target for inflammation and cancer.

**Methods:**

We used molecular docking and experimental approaches to investigate the docking potential and molecular effects of embelin to TACE and human cancer cell characteristics, respectively.

**Results:**

We demonstrate that embelin is a potential inhibitor of TACE. Furthermore, *in vitro* studies revealed that it inhibits malignant properties of cancer cells through inactivation of metastatic signaling molecules including MMPs, VEGF and hnRNP-K in breast cancer cells.

**Conclusion:**

Based on the molecular dynamics and experimental data, embelin is proposed as a natural anti-inflammatory and anticancer drug.

## Background

Inflammation is an immunological process induced by vascular tissues of the body in response to certain external stimuli. It involves various chemical mediators called cytokines that help in the healing of infected tissues. Even though it is a protective response within the body, it may sometimes result in chronic and life threatening effects like rheumatoid arthritis, hay fever, neurodegenerative diseases and cancer [1]. Regulation of cytokines is considered to be a potential therapeutic strategy for the treatment of inflammatory disorders. Many different anti-cytokine approaches including, cytokine neutralization by soluble receptors or activation of anti-inflammatory pathways using monoclonal antibodies are in practice [2]. Tumor necrosis factor alpha (TNF-α), a major immunomodulator and pro-inflammatory cytokine, plays a crucial role in various immunological disorders and inflammations in skin. TNF-α receptors are found in almost all cell types and are known to be involved in several physiological processes. It also leads to multiple inflammatory reactions by inducing the production of secondary cytokines [3]. TNF-α is synthesized as a 223 amino acid long membrane-anchored precursor protein (pro-TNF-α) of 26-kDa. The 17-kDa soluble component of TNF-α is released into the extracellular space by limited proteolysis at the Ala-76 - Val-77 bond [4, 5]. Several different proteases have been found to be involved in this process. Out of these, TNF- α converting enzyme (TACE), a metalloprotease, is considered to be the most efficient enzyme for the proteolytic processing of pro-TNF-α [6]. Since the enzyme plays an important role in converting TNF-α to its soluble form, counteracting the increase in TNF-α concentration in inflammatory disorders by targeting TACE enzyme could provide a potential therapeutic strategy to check inflammation diseases. The role played by TNF-α in the pathophysiology of inflammatory diseases has allowed the development of many new anti-cytokine synthetic drugs that can interfere with excess TNF-α. However, in a study conducted by World Health Organization (WHO), these drugs were associated with drug-related or drug-induced toxic effects, such as, gastric irritation, ulceration, bleeding, renal failure, interstitial nephritis, hepatic failure, headache, thrombocytopenia, hemolytic anemia, asthma exacerbation, skin rashes, angioedema and pruritus [1]. Because of these potential side effects, natural products or herbal drugs are regaining popularity and hence have attracted research attention for solving their mechanism of therapeutic action.

The fruit of *Embelia ribes* Burm (Myrsinaceae) (known as false black pepper in English, Vidanda in Sanskrit and Babrang in Hindi languages) has been in use to treat a variety of gastrointestinal ailments, fever and inflammatory diseases for thousands of years. The active constituent is a quinone derivative, 3-undecyl 2,5-dihydroxy, 1,4-benzoquinone commonly known as embelin, and is isolated from the berries of the plant [7]. It has been shown to possess therapeutic activities like anthelmintic [8], anti-tumor, analgesic [9], anti-inflammatory and anti-diabetic [10], anti-bacterial [11], anticancer [12] and anticonvulsant [13]. The molecular mechanism of such activities of embelin is largely unknown. However, it has been shown that embelin is an inhibitor of X-linked anti-apoptotic protein and also blocks the nuclear factor-kappa B (NF-κB) signaling pathways thus leading to the downregulation of a variety of anti-apoptotic and metastatic gene products [14]. It has also been shown to have *in vivo* anti-inflammatory activity in both acute and choric model of psoriasis or inflammatory skin diseases. It has been reported to reduce TNF-α production in both LPS- and TPA-induced inflammation [7]. In the present study, we first performed molecular dynamic simulations of TACE protein docked with embelin. Based on these data, we investigated the inhibitory effect of embelin on TACE and its downstream signaling involved in cancer cell progression and metastasis. We demonstrate that the embelin-treated human breast cancer cells have reduced levels of TACE and TNF-α. Furthermore, they showed inhibition in growth and cancerous properties including colony forming efficacy, migration and invasion that were mediated by down regulation of MMP-2, MMP-9, VEGF and hnRNP-K proteins.

## Methods

### Protein and ligand preparation

The crystal structure of TACE [PDB ID: 1BKC] was obtained from Protein Data Bank (PDB) [15]. Before docking, ligand present in the structure, obtained from PDB, was deleted. The crystal structure was made clean by removing water molecules. The energy of the protein molecule was minimized by Steepest Descent and Conjugate Gradient method using Accelrys Discovery Studio, the most comprehensive suite for modeling and simulation solutions. The minimization process was carried out using CHARMM force field. The protein was then prepared for docking using Schrödinger’s protein preparation wizard [16]. The protein preparation steps included assigning correct bond orders, addition of hydrogens, creation of disulphide bonds, conversion of selenomethionine to methionine and capping of terminal residues. After the preprocessing and preparation steps, the H-bonds were further optimized.

**Figure 1:**
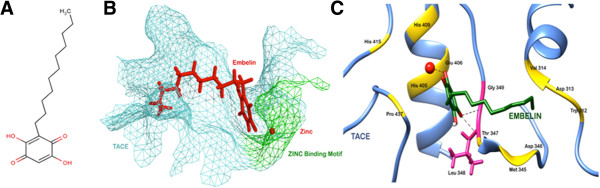
**Docking of embelin to TACE. (A)** Chemical structure of embelin. **(B)** Embelin docked into the active site of TACE. **(C)** Residues of TACE involved in hydrogen bond (pink) and non-bonded (yellow) interactions with embelin.

The ligand molecule, embelin [CID: 3218] was retrieved from NCBI – PubChem Compound Database. Ligand was also prepared using Schrödinger’s LigPrep protocol. It helps in the generation of all possible tautomeric, ionic and stereochemical states of the ligands, followed by their energy minimization. Figure 1A shows the 2D skeleton of the ligand, embelin.

### Prediction of active site

The identification of catalytic residues is a key step in understanding the function of an enzyme. Although some information was available about the active site of TACE from its co-crystallized structure with its inhibitor [15], the active site residue were predicted *in silico* to further validate the available information. Q-site Finder web server was used to predict the most probable active cleft of TACE along with the amino acid residues lining this functionally active site. It uses energy criteria in order to predict the active binding cleft. It calculates the van der Waals interactions of a methyl probe with protein molecule. The probe sites with favorable energy are then clustered based on their spatial proximities. The clusters are ranked according to the total interaction energies, and the cluster with maximum energy is ranked first [17].

### Molecular docking

Glide docking module of Schrödinger [18, 19] was used to investigate the interactions between embelin and TACE. A three dimensional grid was generated with center around the critical residues of TACE, which involve Gly 348, Val 349, His 405, His 409 and His 415 (His residues coordinate with the zinc atom present in the protein molecule). The size of the grid was 20 cubic Å. The docking calculations were performed using the XP (extra precision) mode of Glide. It performs systematic search of conformational, orientation and positional space of docked ligand, discarding unwanted conformations using scoring followed by energy optimization. The conformations are further refined via Monte Carlo sampling of pose conformations. The XP docking score of the complex was coming out to be −9.06 indicating a high binding affinity of the ligand with TACE. The molecular interaction pattern was studied using the Ligplot program [20]. All the docking runs were performed using Intel(R) Core™ 2 Duo CPU, T5870@ 2.00GHz of hp origin, 1.99 GB of RAM.

### Confirmation of docking by AutoDock and Sanjeevni

The docking results obtained from Glide were confirmed using AutoDock Suite 4.0 [21] and ParDOCK [22]. For Autodock, the protein molecule was prepared by adding polar hydrogens for correct ionization and tautomeric states of amino acid residues and non-polar hydrogens were then merged-up. Gasteiger charges and rigid roots were assigned to ligand and 13 bonds were made rotatable. The energy-scoring grid of 60 Å × 60 Å × 60 Å (x, y, z) was prepared incorporating the key residues. The default parameters of Lamarckian genetic algorithm were used as a search protocol for finding the best conformation. To further verify the results, the docked complex was also submitted to ParDOCK which follows Monte Carlo docking protocol [22].

### MD simulations in water

Desmond Molecular Dynamics System [23, 24] with Optimized Potential for Liquid Simulations [25, 26] all atom force field was used to study the dynamic stability of the complex. At first, the complex obtained after molecular docking using Glide XP protocol was solvated in a triclinic periodic box of TIP3 water and then neutralized with appropriate number of counter-ions. The distance between the walls of the box and the complex was kept 10 Å to prevent the interaction of ligand bound protein with its own periodic image. This prepared system was then subjected to energy minimization up to a maximum of 3000 steps using a steepest decent method or until a gradient threshold (25 kcal/mol/Å) was not reached. The equilibrated system was then used to carry out further MD simulations for 10 ns at a constant temperature of 300 K and a constant pressure of 1 atm with a time step of 2 fs. Smooth particle Mesh Edwald method was used to calculate long distance electrostatic interactions. A 9 Å cutoff radius was used for calculating coloumbic short-range interactions. Frames of the trajectory were captured after every 4.8 ps of the time interval.

### Cell culture, treatments, viability and morphological observations

Human breast cancer cell lines, MCF-7 and MDA-MB-231, were obtained from JCRB (Japanese Collection of Research Bio-resources) Cell Bank and cultured in DMEM (Life Technologies, Carlsbad, CA, USA), supplemented with 10% fetal bovine serum and antibiotics at 5% CO_2_ and 95% air in a humidified incubator. Mortalin overexpressing derivatives of MCF-7 and MDA-MB-231 cells were generated by retroviral infections, as described previously [27, 28]. Embelin (Sigma-Alrich, Japan) stock (10 mM in DMSO) was prepared and stored at −20°C. Working concentrations were prepared in DMEM at the time of treatments. Cells were cultured to 60-70% confluency and then treated with embelin. For morphological observations, cells were plated in 6-well plates and treated as indicated. Morphologies of control and treated cells were recorded at 12, 24, 48, 72 and 96 h post-treatments using a phase contrast microscope. Cell viability was determined by MTT assay using 96-well plates. Following incubation with embelin (15 μM)-supplemented medium for 24–72 h, as indicated in the results, cells were incubated with MTT (0.5 mg/ml) for 3 h followed by addition of DMSO (100 μl) to each well. Absorbance was recorded at 550 nm using a multi-well plate reader (Tecan, Switzerland). Data obtained from three independent experiments were analyzed, and the significances were calculated by t-test calculator (GraphPad Software, Inc., CA).

### Colony forming assays

Colony forming ability of cells was examined by plating 500 cells in a 6-well dish. After overnight incubation, the cells were treated with a medium supplemented with embelin. The dish was then left in an incubator for the cells to develop colonies for the next 10–15 days, with a regular change in media every alternate day. Once the colonies were formed, they were fixed in methanol, stained with 0.1% crystal violet, photographed and counted.

### Wound scratch assay

*In vitro* cell migration ability of control and embelin-treated cells was determined by wound scratch assay. Cells were cultured in monolayer, followed by wounding with a 200 μl pipette tip. Cells were washed with PBS to remove off any debris, and then supplemented with embelin containing medium. The time of scratching the wound was designated as 0 h. Cells were allowed to migrate into the wound. The migration ability was recorded at 24 h using a phase contrast microscope at 10 × magnification and quantitated by using the Wimscratch software (Wimasis Image Analysis, Germany).

### *In vitro*chemotaxis assay

Cells (60-70% confluency) were washed with cold PBS, trypsinized, and re-suspended in DMEM-supplemented with 0.5% bovine serum albumin (Sigma) at a cell concentration of 5 × 10^4^ cells/ml. Cells (2.5 × 10^4^) were plated in BioCoat™ Matrigel™ Invasion Chambers (8-mm pore, BD Biosciences), in the presence or absence of embelin, and the invasion assay was performed following the manufacturer’s instructions. Cells that had invaded through the matrigel and migrated through the membrane were extracted with 10% acetic acid, and their absorbance was measured at 590 nm using a Microplate Reader (Tecan, Switzerland).

### Immunoblotting

Cells, after the treatment with embelin, were harvested and lysed by RIPA (RadioImmune Precipitation Assay) Buffer (Thermo Fisher Scientific Inc., IL). 20 μg of protein lysate from control and embelin-treated cells were resolved on 10% SDS-polyacrylamide gels, and transferred to PVDF membrane. The expression level of TACE, MMP-9, MMP-2, VEGF proteins, in response to embelin treatment, was determined by incubating the blots with their specific antibodies followed by probing with respective secondary antibodies. Membranes were probed with anti β-actin antibody (Abcam, Cambridge, UK) as an internal loading control. The pixel calculation of western blots by actin normalization was done using ImageJ software (NIH, MA).

### TACE activity assay

Cells (5 × 10^4^) were plated in 6-well plates. After the cells had fully attached to the substratum, they were incubated with embelin for 24 h, washed with PBS, lysed and their TACE activity was measured following manufacturer’s protocol (Sensolyte, Anaspec Inc., CA). A standard TACE inhibitor, TAPI-0 (10 μM) (Peptides International Inc., Louisville, KY), was used as a control.

### TNF-α ELISA

An ELISA assay for TNF-α was performed using a human TNF-α ELISA kit (Abcam, Cambridge, UK). Briefly, Cells were cultured overnight, followed by treatment with embelin (15 μM) for 24 h. Cell supernatant was then harvested and centrifuged to remove any cell debris. The resultant supernatant was then used for ELISA following the manufacturer’s protocol.

### RT-PCR

RNA was extracted from control and embelin-treated cells using Qiagen RNeasy kit (Qiagen, Limburg, Netherlands). RNA (2 μg) was reverse transcribed to cDNA using the ThermoScript Reverse Transcriptase (Life Technologies) following manufacturer’s protocol. PCR amplifications were performed using equal amount of synthesized cDNA with gene specific sense and antisense primer sets using Phusion High-Fidelity DNA Polymerase (New England Biolabs Inc., MA). PCR amplification conditions were set as initial denaturation (95°C, 5 min) followed by 30 cycles of amplification (95°C for 45 s, 60°C for 1 min and 72°C for 45 s) with final annealing at 72°C for 10 min. Amplified products were resolved on 1% agarose gel, and were visualized by ethidium bromide staining. Quantitation of PCR products was performed with ImageJ and statistical analysis was carried out using Student’s *t* test, wherein *p* values scores ≤0.05 was considered significant. The gene specific primer sequences used as follows: TNF-α: 5′-GGAGAAGGGTGA CCGACTCA-3′ (Sense) & 5′-CTG CCC AGA CTC GGC AA-3′ (antisense); TGF-α 5′-CACACTCAGTTCTGCTTCCA-3′ (sense) & 5′-TCAGACCACTGTTTCTGAGTGGC-3′ (antisense); AREG (Amphiregulin): 5′-GACCTCAATGACACCTACTCTGG-3' (sense) & 5′-AAATATTCTTGCTGACATTTGC-3′ (antisense); Akt: 5'-ATGAGCGACGTGGCTATTGTGAAT-3' (sense) & 5'-GAGGCCGTCAGCCACAGTCTGGATG-3' (antisense); ERK-2: 5'-AAGGTGCCATGGAACAGGCTGT-3' (sense) & 5'-TCCTCTGAGCCCTTGTCCTGAC-3' (antisense); ULBP-2: 5'-CAGAGCAACTGCGTGACATT-3' (sense) & 5'CATGCCCATCAAGAAGTCCT-3' (antisense); CD163: 5'-AGAGGCTGGGGACTGAAAGAA-3' (sense) & 5' GCAGATAACTCCCGCATCCTCCTT-3' (antisense), and GAPDH (internal control) 5′-ACCTGACCTGCCGTCTAGAA-3′ (sense) & 5′-TCCACCACCCTGTTGCTGTA-3′(antisense).

### Immunofluorescence

For immunofluorescence study, cells were cultured on coverslips placed in 12-well dish. After overnight incubation, cells were treated with embelin for 24 h, washed with cold PBS and fixed with methanol: acetone (1:1) for 5 min. Fixed cells were washed twice with 1 X PBS, permeabilized using 0.5% Triton X-100 in PBS for 10 min, and blocked using 2% BSA in PBS for 15 min. Coverslips containing cells were incubated with antibodies against TACE, MMP-9, MMP-2, VEGF (Santa Cruz Biotechnology Inc., Texas), hnRNP-K (Cell Signaling Technology Inc., MA) proteins for 2 h at room temperature, washed thrice with 0.2% Triton X-100 in PBS followed by incubation with Alexa Fluor conjugated secondary antibodies. After further washings with 0.2% Triton X-100 in PBS, cells on coverslips were mounted and visualized under Carl Zeiss microscope (Axiovert 200 M).

## Results

### Identification of the active catalytic cleft in TACE protein

The pre-processed structure of TACE was submitted to Q-site Finder server. Based on the interactions of probe with protein molecule it returned ten energetically favored clusters. The individual probe sites relate most closely to the favored high-affinity binding sites on the protein surface and are the locations where a putative ligand could bind and optimize its van der Waals interaction energy [17]. The topmost site in the results obtained had a volume of 571 cubic Å. It included approximately 27 residues, namely Gly 346, Thr 347, Leu 348, Gly 349, Leu 350, Ala 351, Asn 389, Tyr 390, Lys 392, Thr 393, Ile 394, Leu 395, Glu 398, Ala 399, Leu 401, Val 402, His 405, Glu 406, His 409, His 415, Tyr 433, Val 434, Met 435, Tyr 436, Pro 437, Ile 438, Ala 439, Val 440 and Ser 441. The grid generated while molecular docking was made to cover all of these residues that constituted the active site.

### Flexible docking of embelin into the functional cavity of TACE

A possible mode of action proposed here to substantiate the role of embelin in reducing the levels of TNF-α in inflammation is by interaction of ligand with the key residues of TNF-α converting enzyme (TACE). Chemical structure of embelin is shown in Figure 1A. The Glide XP score for binding of embelin to TACE was −9.06, which indicated a high affinity of embelin for TACE protein. As shown in the Figure 1B, the ringed structure forming the head of the ligand got buried into the active pocket of TACE, while the long hydrophobic twisted tail was also found to interact closely with the small groove in the protein, as shown by the mesh representation. The active cleft of TACE has catalytic zinc residing at its center, penta-coordinated by three imidazole N_2_ atoms of His 405, His 409 and His 415 [15]. The active center of TACE has also been reported to possess remarkable similarity with other zinc metalloproteases (MMPs) including a conserved amino acid sequence-HExGHxxGxxH- [29]. This motif in TACE stretches from residue number 405 to 415. In general reaction mechanism, the zinc ion assumes a quasi penta-coordinated state after dissociating from the histidine residue of this conserved zinc-binding motif. This change in state causes the polarization of oxygen atom of the glutamic acid that lies close to the scissile bond of the substrate, thereby acting as a reversible electron donor. This forms an oxyanion transition state. At this stage, the water molecule acts on the dissociated scissile bond and completes the hydroxylation of the substrate [30]. The binding of embelin to TACE was characterized by H-bonds formed with two of the critical residues namely Leu 348 and Gly 349 as illustrated in Figure 1C. Previous studies have reported that these two residues play an important role in the catalytic activity of the protein [31]. The length of the H-bonds was 2.80 and 3.06 Å respectively. Embelin was also found to form a coordinate bond with the zinc atom. Many residues of this conserved zinc-binding motif were seen to interact with the ligand. These included His 405, Glu 406 (which acts as a general base during catalysis), His 409 and His 415. Along with these residues, Trp 312, Asp 313, Val 314, Asp 344, Met 345, Thr 347 and Pro 437 lining the inner surface of the active site were also showing hydrophobic and van der Waals interactions with the docked embelin.

### Mimicking the *in vivo*conditions using molecular dynamics simulations

**Figure 2:**
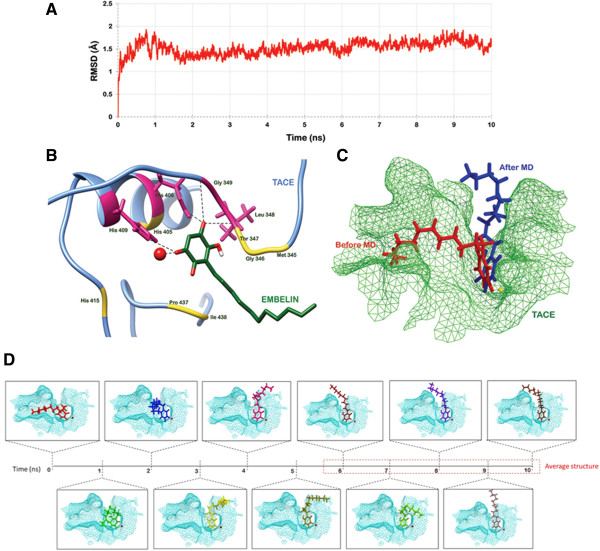
**Kinetic of embelin docking to TACE. (A)** Trajectory showing root mean square deviation in the conformation of TACE backbone in reference to the docked complex over the entire simulation run. **(B)** Molecular interaction pattern between TACE and embelin after MD simulations. **(C)** Difference in the binding pose of embelin before (red) and after (blue) MD simulation. **(D)** Changes observed in the docking conformation of embelin during the simulations of 10 ns duration.

It is important to study the protein ligand interaction in dynamic motion. Desmond Molecular Dynamic System was used for 10 ns simulation of the complex (Figure 2). Figure 2A shows the RMSD of backbone of TACE protein calculated in reference to the first frame over the entire simulation trajectory. As observed from the RMSD graph, the protein deviated up to 2 Å in first half of the simulation run after which it acquired quite a stable state. Figure 2D shows the change in the orientation of the bound embelin with progression in the simulation time. The ligand did not show significant variation in the time frame of 7 to 10 ns. So, a representative average structure was generated for this time period. The protein backbone was also most stable in this interval of time as depicted by the RMSD curve. The residues of TACE that were involved in hydrogen bonding with the embelin molecule were investigated using the average structure. A total of 34 hydrogen bonds were formed during the entire simulation run of 10 ns. The residue pairs with high occupancy were the ones that persisted for more than half of the run and hence were responsible for the stable binding of embelin within the active site of TACE. Table 1 lists all the H bonds with their occupancy. Figure 2B illustrates the binding pattern of embelin with TACE in its dynamically stable conformation, represented by the computed average representative structure. It was observed that the two H bonds found subsequent to docking persisted even after the 10 ns simulation run with occupancy of 92.81 and 73.54%. Apart from these bonds, 2 new H bonds of 2.65 and 3.05 Å were formed involving Glu 406 and His 409 respectively. The bond of 2.65 Å between Glu 406 and embelin had the maximum occupancy, of 96.74%. Even though a bond between Gly 346 and embelin also was observed with occupancy of 43.43%, it did not appear in the structure representing the most stable time frame. The data indicated that this bond was lost when the energetically favored conformation achieved by the docked complex. Multiple hydrophobic and van der Waals interactions further stabilized the binding of embelin to TACE. The residues which participated in these interactions included Met 345, Gly 346, Thr 347, His 405, His 415, Pro 437 and Ile 438. Figure 2C shows shift in orientation of the ligand within the catalytic cleft of TACE after attaining a stable state. The position of the docked ligand changed significantly during MD simulation. This shift was monitored in reference to the stoichiometry obtained after molecular docking.

**Table 1:** **List of residues pairs participating in hydrogen bond formation during the entire MD simulation run**

S. No.	Donor	Acceptor	Occupancy (%)
1	**Leu 348 (M)**	**Embelin**	**92.81**
2	**Gly349 (M)**	**Embelin**	**73.54**
3	**Gln406 (S)**	**Embelin**	**96.74**
4	Embelin	Gly346(M)	43.43
5	Thr347 (S)	Embelin	1.82
6	His405 (S)	Embelin	3.79
7	Embelin	Gly349 (M)	4.03
8	Val314 (M)	Embelin	0.10
9	Embelin	Val314 (M)	0.10
10	Embelin	Thr347 (S)	1.87
11	Embelin	Glh406 (S)	13.71
12	Embelin	Pro437 (M)	21.33
13	Embelin	Asp344 (M)	0.29
14	Leu348 (S)	Embelin	4.94
15	Thr347 (M)	Embelin	3.26
16	Embelin	Asp313 (S)	0.10
17	**His409 (S)**	**Embelin**	**41.56**
18	Met345 (S)	Embelin	0.10
19	Embelin	Met345 (S)	0.14
20	Embelin	Ile438 (M)	0.05
21	Val314 (S)	Embelin	0.10
22	Embelin	Val314 (S)	0.10
23	Embelin	Met345 (M)	5.90
24	Embelin	His405 (S)	0.14
25	Pro437 (S)	Embelin	6.14
26	Embelin	Leu348 (M)	0.77
27	Embelin	Leu348 (S)	0.43
28	Embelin	His409 (S)	0.05
29	Embelin	Tyr436 (S)	0.34
30	Tyr436 (S)	Embelin	0.05
31	Embelin	Tyr390 (S)	0.24
32	Embelin	Pro437 (S)	0.05
33	Tyr390 (S)	Embelin	0.05
34	Embelin	Thr347 (M)	0.05

(M) Main chain, (S) Side Chain. The residues in bold format are the ones that were involved in H-bond formation in the representative average structure of TACE-embelin complex, obtained subsequent to molecular dynamics simulations.

### Embelin-treated breast cancer cells showed reduction in TACE and inhibition of cancer cell growth and metastasis

**Figure 3:**
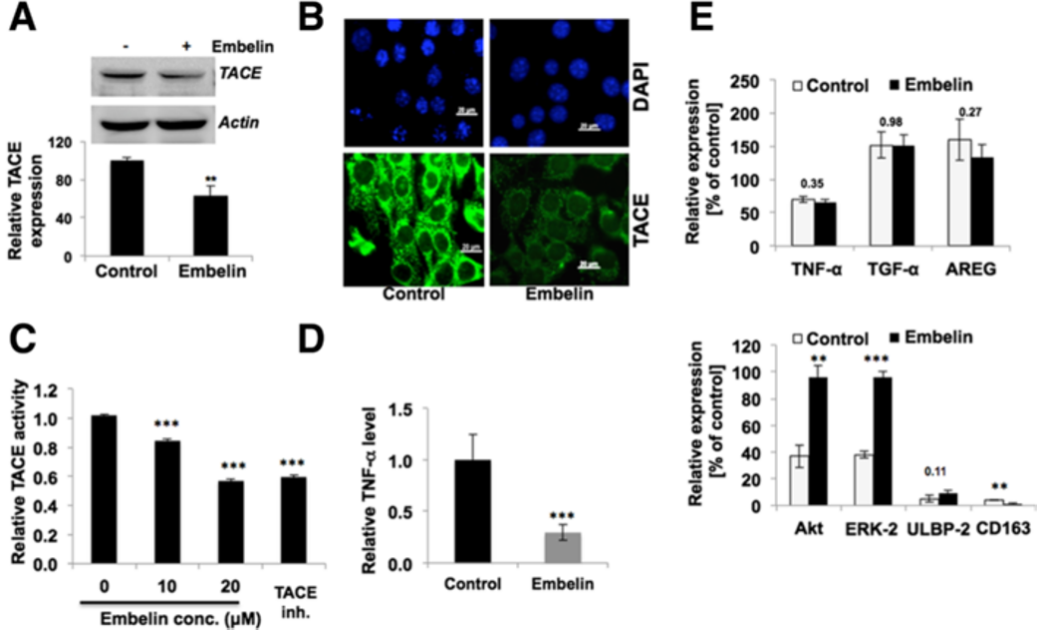
**Effects of embelin on TACE expression and activities. (A)** Expression of TACE in control and embelin (15 μM)-treated MCF7 cells as detected by western blotting. Actin was used as an internal control. **(B)** Immunostaining of TACE in control and embelin-treated MCF-7 cells. **(C)** TACE activity in control and embelin-treated cells; TACE inhibitor, TAPI-0 (10 μM), was used as a control. **(D)** TNF-α expression in control and embelin-treated cells. **(E)** RT-PCR analysis of TACE-effectors showing no change in TNF-α, TGF-α and ARFG (upper panel); Akt, Erk-2 and ULBP-2 showed increase, and CD163 showed decrease in embelin-treated cells.

In order to validate the molecular dynamics results of docking embelin to TACE, we performed experiments using two human breast cancer cell lines, MCF7 and MDA-MB-231. Cells treated with embelin showed decline in their viability with IC_50_ dose of 15–20 μM. We next treated the cells with 15 μM embelin for 48 h and examined their TACE expression level by western blotting and immunostaining. As shown in Figure 3A and B, embelin-treated cells showed statistically significant (p < 0.01) decrease in TACE expression in multiple independent experiments. Similar results were seen in immunostaining of TACE in control and embelin-treated cells. Furthermore, the activity of TACE, as quantitated by measuring the TACE-dependent fluorescence released by quenched QXL^TM^520/5-FAM FRET substrate (Sensolyte, Anaspec Inc., CA), decreased (~50%) in cells treated with 20 μΜ of embelin (Figure 3C). Consistent with this, the level of TNF-α, a downstream effector of TACE, also decreased (4-fold) in embelin-treated cells as compared to the untreated controls (Figure 3D). We also performed RT-PCR analysis in control and embelin-treated cells for down-stream effectors of TACE, including TNF-α, TGF-α, AREG, Akt, Erk-2, ULBP-2 and CD163. Whereas no difference was observed in TNF-α, TGF-α and AREG; there was an increase in Akt, ERK-2 and ULBP-2, and decrease in CD163 (Figure 3E).

**Figure 4:**
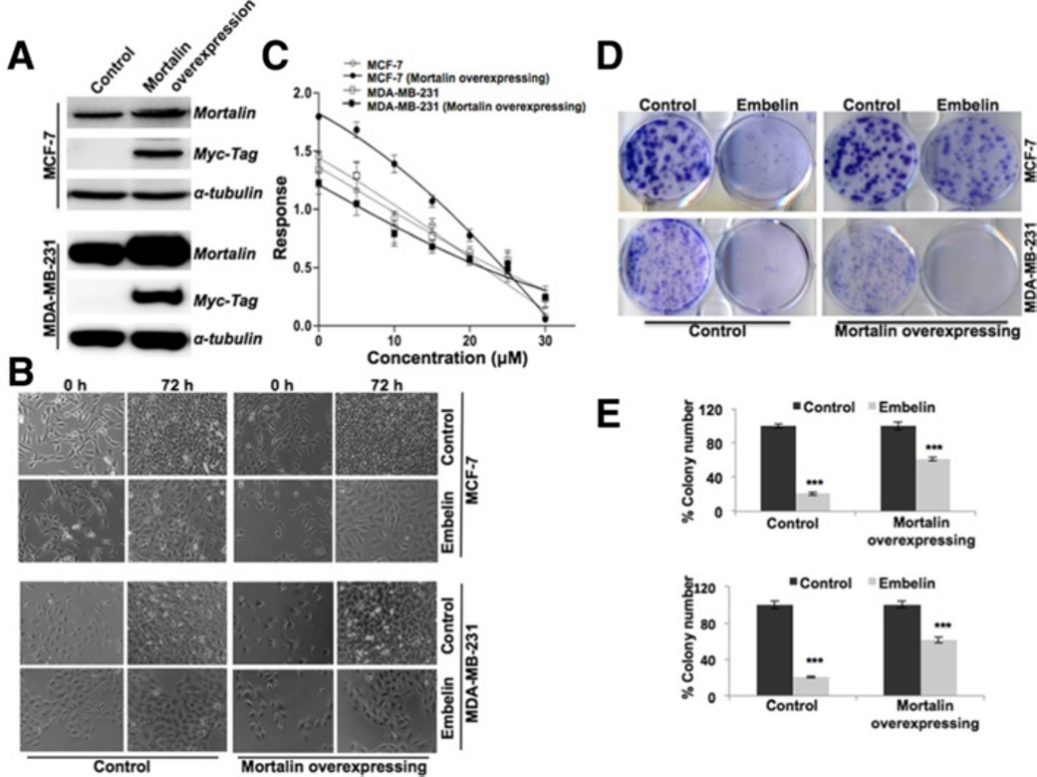
**Effects of embelin on proliferation and migration of breast cancer cells. (A)** Expression of mortalin in mortalin-myc transduced metastatic cells detected by western blotting with anti-myc and anti-mortalin antibodies. **(B)** Morphology of embelin-treated control and mortalin-overexpressing metastatic cells indicative of growth arrest. **(C)** Viability of human breast cancer cells (MCF7 and MDA-MB-231) and their mortalin-overexpressing derivatives. The 4-parameter logistic curve is plotted using Prism 6 software (GraphPad Software, Inc., CA). **(D)** Colony forming efficacy and quantitation **(E)** from three independent experiments is shown. ^***^p < 0.001.

We have earlier reported that the overexpression of mortalin/mtHsp70 in cancer cells contributes to their malignant properties, including increased colony forming efficacy, migration and invasion [27, 28]. In order to investigate the effect of embelin on cancer cell metastasis, we next generated mortalin-overexpressing metastatic derivatives of MCF7 and MDA-MB-231 cells. Expression of myc-tagged exogenous expression of mortalin was confirmed by western blotting with anti-myc tag antibody (Figure 4A). Morphological observation of cells in either control or embelin-supplemented medium revealed their growth arrest in the latter (Figure 4B). As shown in Figure 4C, we found that the embelin was cytotoxic to both MCF7 and MDA-MB-231 cells, and their metastatic derivatives in equivalent doses (15 μM), suggesting its potency for the treatment of metastatic cancers. Embelin caused significant reduction in colony forming efficacy (CFE); 80% and 40% reduction in MCF7 cells and its metastatic derivatives, respectively. Embelin-treated MDA-MB-231 cells revealed about 90% reduction in CFE in control (parent) cells and 80% in metastatic derivatives (Figure 4D and E) supporting that embelin could be a potent drug to treat metastatic cancers.

**Figure 5:**
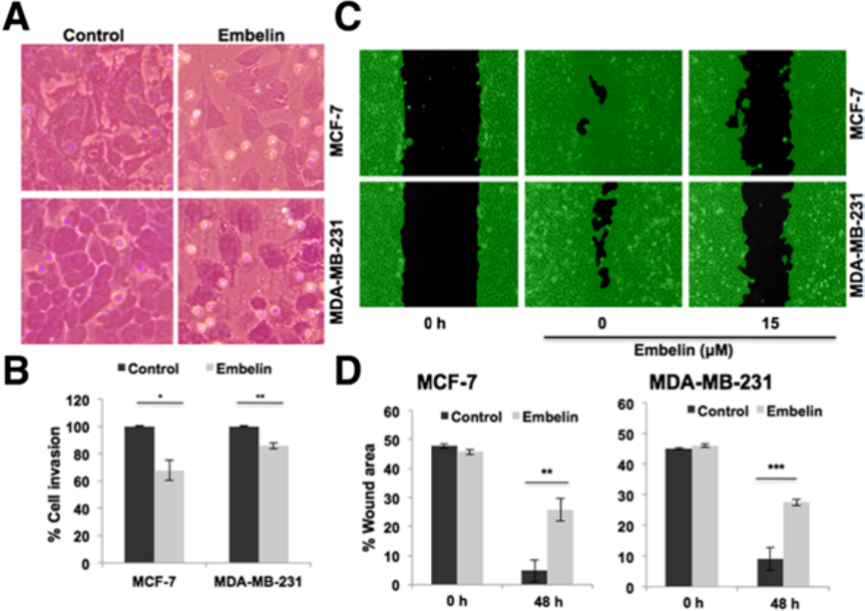
**Effects of Embelin on invasion capacity of breast cancer cells. (A)** Cell invasion assay in control and embelin-treated cells. **(B)** Quantitation from three independent experiments showing 20-30% decrease in invasion capacity; ^*^p < 0.05 and ^**^p < 0.01. **(C)** Wound-scratch assay of the control and embelin-treated cells. **(D)** Quantitation from three independent experiments showing 20-30% decrease in the migration capacity of cells. *p < 0.05 and ^**^p <0.01.

**Figure 6:**
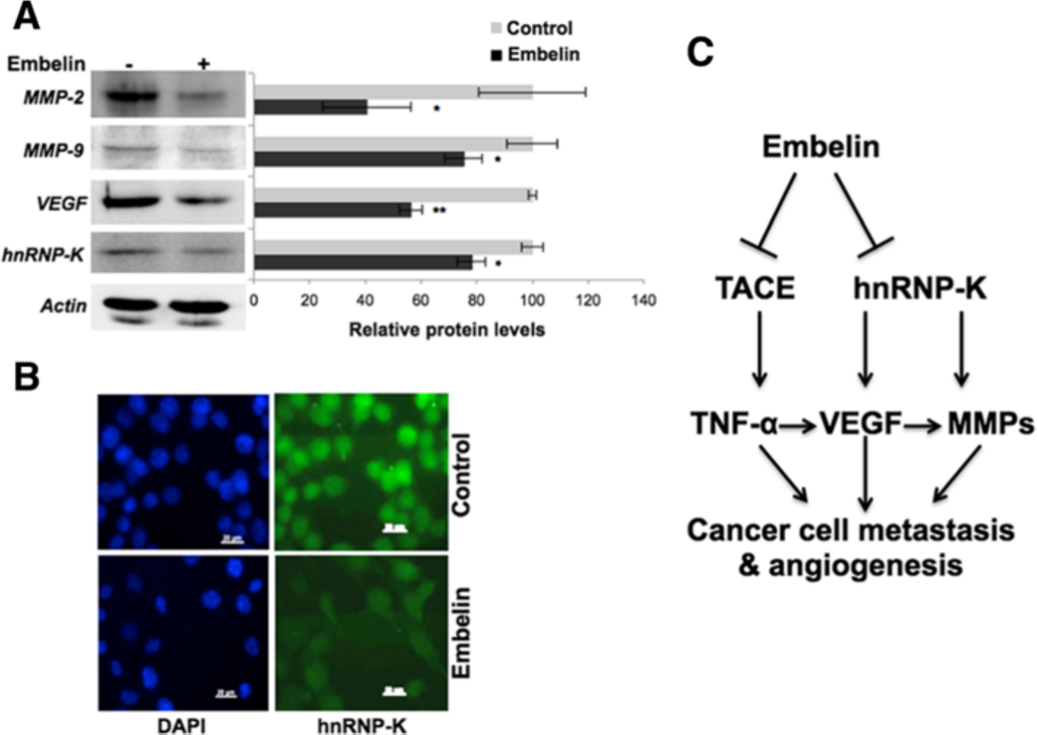
**Effects of embelin on metastasis-mediating proteins.** Western blot analyses of proteins involved in cancer cell migration. **(A)** MMP-2, MMP-9, VEGF and hnRNP-K decrease in embelin-treated MCF-7 cells. The pixel calculation of western blots by actin normalization was done using ImageJ software (NIH, MA). **(B)** Decrease in the expression of hnRNP-K, as detected by immunostaining. **(C)** Schematic representation of possible action of embelin on cancer cell metastasis as based on the present study.

In order to evaluate the effect of embelin on cancer metastasis, we performed cell migration and wound scratch assays in control and embelin-treated cells under the conditions (15 μM embelin treatment for 10–20 h) when their growth, *per se*, was not affected. We found that the invasion of both MCF7 and MDA-MB-231 cells through matrigel was inhibited in embelin-treated cultures (Figure 5A and B; quantitation) under the conditions when the cell number was not altered (data not shown). Visual examination of cell migration by wound-scratch assays also revealed slower migration of cells in the wound area in MCF-7 and MDA-MB-231 cells (Figure 5C and D). In light of this data, we next examined the expression of metastatic markers including MMP-2, MMP-9 and VEGF in control and treated cells and found reduction (60%, 25% and 48%, respectively) in their level of expression (Figure 6A). We, next, examined the expression status of an upstream regulator, hnRNP-K that has been shown to play critical role in cell migration. We found that in response to embelin treatment there was a significant reduction in hnRNP-K (Figure 6A and B), supporting its potency for treatment of metastatic cancer.

## Discussion

In the present study, we investigated the molecular mechanism of the effect of embelin on TACE and cancer cell characteristics. We found that embelin docks into the active site of TACE that contains zinc atom coordinated by a conserved zinc binding motif (405-HexGHxxGxxH-415) [15] as shown by the analysis of three dimensional structure of TACE by Q-site Finder server. Apart from this conserved motif, the binding pocket also had Lys 348 and Gly 349, which are considered to be important for the activity of this enzyme [31]. The predicted pocket also coincided with the binding site of the ligand present in the co-crystallized structure obtained from PDB (1BKC). The docking score of 3D structure of embelin with the above mentioned active site of TACE was −9.06 suggesting high binding affinity of embelin for TACE. It showed molecular interactions with histidine and glutamate residues, which play an important role during the proteolytic reaction process. Furthermore, embelin also showed bonding with the zinc atom. Taken together, these interactions of embelin and TACE were expected to interfere with the interaction of substrate to the active site of TACE, hence consolidating the idea of embelin as TACE inhibitor.

The dynamics of the docked complex were then studied to analyze its stability inside the bodily conditions. A simulation length of 10 ns was used in the study to allow rearrangement of the ligand bound protein molecule to find its stable binding mode. The RMSD trajectory was analyzed to comment on the stability of the docked complex. The curve did not deviate much after 6 ns, which indicated that the complex has reached its energetically favored conformation. A structure representing the most stable time frame was used to examine the molecular interaction pattern in the ligand bound protein complex. Various non-covalent interactions including H-bonds, hydrophobic interactions and van der Waals contacts were responsible for the stable interaction of embelin with TACE. The ringed structure of embelin occupied the same groove over the entire simulation run, whereas the long hydrophobic tail found a new groove for itself. Some new residues were interacting with embelin because of this shift in the position of the tail region. The dynamic stability of ligand during majority of the simulation time and its interactions with active site key residues of the enzyme plausibly conclude the mode of action of embelin on inhibition of TACE and it’s well known anti-inflammatory activity.

We next investigated the effect of embelin on TACE expression level in breast cancer cell lines. The data revealed decrease in TACE and growth arrest of cancer cells in response to embelin treatment. TACE processes precursor TNF-α to its bioactive 17-kDa protein with high specificity and efficacy [32]. The latter is not only an important mediator of inflammatory phase of wound healing, but also a key regulator matrix re-modeling, angiogenesis and tumor metastasis. Embelin-treated cells showed suppression of TACE activity, supported by analysis of downstream effectors of TACE. Consistent with other reports, TACE inhibition was endorsed by transcriptional activation of Akt [33, 34], Erk-2 [35] and ULBP-2 [36] and repression of CD163 [37]. On the other hand, level of expression of TNF-α, TGF-α and AREG transcripts did not show any difference in control and embelin-treated cells, suggesting that the inhibition of TACE in response to the treatment with embelin may operate at protein level and the increase in Akt and Erk-2 represent adaptive feedback response of cells [33–38].

Tumor microenvironment, tumor-associated macrophages and cytokines in particular, have been established to play key role in progression, metastatic spread of breast cancer and angiogenesis that are indeed the major cause of therapeutic failure. Macrophages are activated by cytokines to secrete angiogenic factors including vascular endothelial growth factor (VEGF) that contribute to cancer cell aggressiveness. Since the release of cytokines from tumor cells is mediated by their ectodomain shedding by TACE, specific inhibitors of TACE have been in clinical trials as therapeutic drugs for aggressive and advanced metastatic cancers. Based on our above findings on the targeting and inhibition of TACE by embelin, we investigated whether embelin could inhibit metastasis of breast cancer cells. Consistent with our prediction, metastatic derivatives of the cell lines, generated by mortalin overexpression, were also found to undergo growth arrest and decrease in the malignant characteristics including cell migration and invasion in the presence of embelin. As shown in the schematic diagram in Figure 6C, TNF-α has been shown to stimulate synthesis and secretion of active MMPs [33]. We found that MMP-2 and MMP-9 were significantly decreased in embelin-treated cells suggesting its anti-metastasis potential. Furthermore, upstream regulators of MMPs, hnRNP-K and VEGF [39–42] were also downregulated in embelin-treated cells suggesting that embelin might block multiple cancer cell metastasis signaling pathways (Figure 6C). Although the molecular intricacies of these regulations warrant further studies, the present study provided evidence that the anti-inflammatory and anti-metastasis activities of embelin may be mediated by its binding to TACE.

## Conclusion

Molecular docking and experimental data demonstrated that embelin is a potential inhibitor of TACE. Furthermore, it inhibited malignant properties of breast cancer cells through inactivation of metastatic signaling molecules including MMPs, VEGF and hnRNP-K, and hence proposed it as a natural anticancer drug.
